# Current Management and Volar Locking Plate Fixation with Bone Cement Augmentation for Elderly Distal Radius Fractures—An Updated Narrative Review

**DOI:** 10.3390/jcm12216801

**Published:** 2023-10-27

**Authors:** Ting-Han Tai, Po-Jui Chu, Kuan-Yu Lu, Jeffrey J. Wu, Chin-Chean Wong

**Affiliations:** 1 Department of Orthopedics, Taipei Medical University Shuang Ho Hospital, New Taipei City 235041, Taiwan22267@s.tmu.edu.tw (P.-J.C.);; 2 Department of Primary Care Medicine, Taipei Medical University Shuang Ho Hospital, New Taipei City 235041, Taiwan; 3 Department of Orthopedics, School of Medicine, College of Medicine, Taipei Medical University, Taipei 110301, Taiwan; 4 Research Center of Biomedical Devices, Taipei Medical University, Taipei 110301, Taiwan; 5 International PhD Program for Cell Therapy and Regenerative Medicine, College of Medicine, Taipei Medical University, Taipei 110301, Taiwan

**Keywords:** elderly distal radius fracture, management, volar plate, bone cement augmentation

## Abstract

Distal radius fractures (DRFs) are the most common among all kinds of fractures with an increase in incidence due to the rapidly expanded size of the elderly population in the past decades. Both non-surgical and surgical treatments can be applied for this common injury. Nowadays, more and more elderly patients with DRFs undergo surgical treatments to restore pre-injury activity levels faster. However, optimal treatment for geriatric DRFs is still debated, and careful evaluation and selection of patients are warranted considering clinical and functional outcomes, and complications following surgical treatments. Furthermore, osteoporosis is a predominant factor in elderly DRFs mostly deriving from a low-energy trauma, so many treatment modalities are developed to enhance better bone healing. Among various options for bone augmentation, bone cement is one of the most widely used measures. Bone cement such as calcium phosphate theoretically improves fracture stability and healing, but whether the elderly patients with DRFs can significantly benefit from surgical fixation with bone cement augmentation (BCA) remains controversial. Hence, in the present review, the latest literature regarding current concepts of management and evidence about volar locking plate fixation (VLPF) with BCA for elderly DRFs was searched in MEDLINE, Embase, Cochrane Central Register of Controlled Trials, and Web of Science; out of >1000 articles, full texts of 48 and 6 articles were then examined and analyzed separately for management and VLPF with BCA for elderly DRFs. We aim to provide the readers with updates concerning the above issues.

## 1. Introduction

The distal radius fracture (DRF) is the second most common fracture in the elderly aged 65 years or greater, accounting for up to 18% of all fractures in this population [1,2,3]. The incidence of DRFs increases as the population ages in the past several decades [1,4]. Risk factors associated with this common injury include white race, female gender, and osteoporosis [2,3]. Non-displaced DRFs can be treated non-surgically through closed reduction (CR) and immobilization with a splint or cast [5,6,7,8], whereas displaced ones might warrant surgical treatments such as closed reduction and external fixation (CREF), percutaneous Kirschner wire (K-wire) fixation, and open reduction and internal fixation (ORIF) [5,6]. However, whether the elderly patients with unstable displaced DRFs should undergo surgical treatments has remained controversial despite a marked increase in the surgical rate like that of the non-elderly in the past decades [4,9,10,11,12,13,14]. The complication rate appears to be higher in those receiving surgical treatments, and the differences of clinical and functional outcomes are insignificant between non-surgical and surgical treatments [10,11,12,13,14]. Consequently, in the latest American Academy of Orthopedic Surgeons (AAOS)/American Society for Surgery of the Hand (ASSH) evidence-based clinical practice guideline (CPG) on DRFs, the workgroup indicated that surgical treatments for geriatric patients do not lead to improved long-term patient-reported outcomes (PROs) compared to non-surgical treatments [15].

ORIF using a volar locking plate (VLP) has been proved to provide good functional outcomes and to be cost-effective for both intra-articular and extra-articular DRFs [16,17]. In the elderly, volar locking plate fixation (VLPF) also seems to be a safe and effective procedure that facilitates early recovery of hand and wrist functions [18], and it leads to a similar complication rate to that from younger patients [19]. Nonetheless, except for providing better functional and radiographic assessment outcomes, VLPF is unable to superiorly improve the Disabilities of the Arm, Shoulder, and Hand (DASH) score and complication rates compared to other conservative treatments [11,12,19,20].

VLPF with bone cement augmentation (BCA) is a promising technique since it theoretically increases the stability by compensating for the shortcomings of VLPF alone in osteoporotic bones [21,22,23]. There have been few studies investigating this combination modality. One small-scale randomized–controlled trial (RCT) and two retrospective comparative studies found that BCA on VLPF offers no additional benefits in comparison with VLPF alone [24,25,26], yet two biomechanical studies reported improvement in biomechanical properties from augmented VLPF [27,28].

In the present review, we searched and reviewed the latest evidence, especially clinical trials within 5 years, in terms of current concepts of management and the use of VLPF with BCA for elderly DRFs. Databases including MEDLINE, Embase, Cochrane Central Register of Controlled Trials, and Web of Science were searched for the related literature using different combinations of keywords including elderly, geriatric, distal radius fractures, complication, rehabilitation, osteoporosis, volar plates, bone cement, and augmentation. The two co-first authors then independently screened titles and abstracts of probably related literature and identified articles that are within the scope of this review. After excluding unrelated studies comprising most of the searched literature (>1000), full texts of 48 and 6 articles were examined and analyzed for management and VLPF with BCA for elderly DRFs separately. Moreover, we provided our clinical experiences and proposed future research directions for geriatric DRFs. Our aim is that this article could provide the readers with an updated and comprehensive understanding of elderly DRFs.

## 2. Management of Elderly DRFs

According to the 2020 AAOS/ASSH CPG on DRFs, even though surgical fixation is not conclusively suggested for elderly patients, high-functioning patients with high functional demands despite an age greater than 65 years may benefit from surgical fixation [15]. Therefore, in this section, we reviewed and summarized the latest literature pertaining to the comparison between non-surgical and surgical treatments, outcomes, complications, rehabilitation, and considerations in osteoporosis for elderly DRFs.

### 2.1. Non-Surgical Treatment

Applying a splint or cast after CR is one of the most common methods for immobilization in the treatment of DRFs [5,6,7,8]. A prospective multicenter RCT comparing short-arm and long-arm plaster casts for the treatment of stable DRFs in patients older than 55 years found that a short-arm cast is as effective as a long-arm cast; furthermore, it is more comfortable and introduces less restriction on daily activities [29]. Traditionally, wrists have been placed in the volar-flexion and ulnar-deviation (VFUD) position in DRFs. Recently, there has been a trend towards using a functional-position cast (FC). A prospective RCT evaluating the functional results and costs of the above 2 casting positions in patients aged 65 years and older with DRFs found that VFUD is not superior to FC; further cost analyses reveal that overall costs from VFUD are nearly twice those from FC [30]. As to the period of casting time, an RCT compared the functional and radiographic outcomes following treatment of DRFs in a cast for 4 and 6 weeks in the elderly; The Visual Analogue Scale (VAS) pain score, Mayo Wrist Score (MWS), and VAS activity score are similar between the 2 study groups, and there is no significant between-group difference in terms of radial inclination, union rate, radial height, or bone union [31]. In comparison with surgical treatments, cast immobilization (CI) is non-inferior to VLPF after 1 year in patients aged 65 years and older [32].

Probiotic treatment is another optional non-surgical supplementary treatment for elderly DRFs since it has been shown to improve bone formation, increase bone mass density, and prevent bone loss [33]. Lei et al. found that the DASH score, pain, complex regional pain syndrome (CRPS) score, wrist flexion, and grip strength from patients with DRFs receiving probiotics exhibit a significantly faster pace of improvement than those on placebo, with treatment outcomes of patients receiving Lactobacillus casei Shirota (LcS) at month 4 at comparable levels with those of patients receiving placebo at month 6 [34]. Similarly, Zhang et al. also concluded that oral administration of LcS dramatically accelerates hand function recovery in senior patients with DRFs [35].

### 2.2. Surgical Treatment

There are several options in surgical treatments for elderly DRFs, including CREF, percutaneous K-wire fixation, and ORIF using a volar or dorsal locking plate or a dorsal bridge plate [5,6]. In displaced DRFs, the Combined Randomised and Observational Study of Surgery for Fractures in the Distal Radius in the Elderly (CROSSFIRE) Study Group conducted an RCT and found no superior improvement in wrist pain or function at 12 and 24 months from VLPF over CR [36,37].

Specifically for dorsally displaced DRFs (i.e., Colles fractures) in the elderly, Tahir et al. compared closed reduction and cast immobilization (CRCI) to anterior plating and found that the Patient-Rated Wrist Evaluation (PRWE) score at 12 months is not significantly different between the 2 groups; however, radiologic outcomes and complication rates are worse in the CRCI group [38]. On the other hand, an RCT implied that the PRWE scores, DASH scores, and grip strength are better from VLPF compared to a plaster splint at 3 and 12 months, and the complication rates are similar [39]. Another RCT also concluded that VLPF results in less long-term disabilities compared to non-surgical treatments for severely displaced DRFs in patients aged above 70 years [40].

As to delayed surgery after primary non-surgical treatments, Sirniö et al. found that early palmar plating results in better 2-year functional outcomes for patients aged above 50 years, and delayed surgery in case of secondary displacement is not beneficial in terms of function [41].

For intra-articular DRFs in the elderly, Martinez-Mendez et al. found that VLPF leads to better patients’ quality of life, functional outcomes, and radiologic parameters than CRCI [42]. As to unstable DRFs, in an RCT by the Wrist and Radius Injury Surgical Trial (WRIST) Group, the authors found no clinically meaningful PRO difference 24 months after injury across VLPF, external fixation (EF), percutaneous pinning, and CRCI, with little changes between 12 and 24 months [43]. Thorninger et al. also found that VLPF does not superiorly improve the functional outcomes after 5 weeks, 6 months, and 12 months compared to casting, and complication rates are similar [44]. However, modified K-wire fixation might be a safe, effective, rapid, and minimally invasive surgical option owing to higher patient satisfaction, shorter operation time, and a lower complication rate compared to ORIF [45].

Despite growing evidence for less invasive treatments in elderly DRFs, a Finnish nationwide study discovered that plate fixation has almost completely replaced both EF and percutaneous pinning [46]. However, Heng et al. claimed that surgical fixation of DRFs in appropriately selected patients in the super-elderly population yields good functional outcomes [47], and Huang et al. also found that VLPF provides better radiologic outcomes, wrist supination, and lower complication rates than EF in the geriatric population aged over 80 years [48]. Compared to those in middle-elderly patients, comparable clinical and radiographic outcomes are found in DRFs treated with VLPF in super-elderly patients [49]. Additionally, a recent meta-analysis demonstrated that VLPF provides measurable benefits in grip strength and fewer complications to those aged 60 years and over [50].

The application of minimally invasive approaches with advantages of both non-surgical and surgical treatments for elderly DRFs might make the best of both worlds. The IlluminOss^®^ photodynamic bone stabilization system is a novel minimally invasive, percutaneous intramedullary polymeric osteosynthesis technique that allows clinicians to repair bone fractures using a light-curable polymer contained within an inflatable balloon catheter, offering a new treatment option for osteoporotic long bone fractures [51,52]. In elderly DRFs, this system is preliminarily proved to be a feasible option with seemingly good clinical and functional outcomes [53,54].

### 2.3. Post-Treatment Outcomes

In a systemic review by Fogel et al. regarding outcome metrics following the treatment of DRFs in patients aged above 50 years, physical examination findings and radiographic measures are reported in 70% and 74% of studies despite known limitations of these metrics. PRO measures are used to assess outcomes in 74% of studies, among which only the DASH score is used in greater than half of the studies (57%). Pain scores are assessed in 39% of studies, and complications are in only 26% [55] (Table 1).

Pain at enrollment, education, physiological instead of chronological age, and number of comorbidities are reported to be outcome predictive factors for elderly DRFs [64,65]. A dorsal tilt greater than 5 degrees, ulnar positivity greater than 2 mm at baseline, after treatment, and at the final follow up, and persistent articular gaps/steps greater than 2 mm after treatment are also associated with worse PROs [66,67]. However, treatment types and precise anatomic restoration do not affect the 12-month outcomes [64,67,68]. Pre-injury activity levels are also predictive of PROs and functional outcomes following DRFs in patients aged 60 years and older, and VLPF is preferable to other treatments for highly active patients because of the greater PROs, early mobility, and lower risk of hardware infection [69]. As to the correlation between functional outcomes and patient satisfaction, Chung et al. found that the optimal cut-off points to distinguish satisfaction from dissatisfaction occur when patients recovered 59% of hand strength and 79% of wrist motion, which might infer continuing therapy to increase functional gains beyond this point without additional patient-reported gains [70].

A recent network meta-analysis of 23 RCTs comprising 3054 participants found that the DASH and PRWE scores from ORIF are significantly lower compared to casting, implying that ORIF may be associated with clinically significant improvements in short-term recovery. In the intermediate term, ORIF also has lowered DASH and PRWE scores. One-year complication rates are comparable among all treatments [71]. However, both the QuickDASH and PRWE demonstrate ceiling effects with some patients achieving ceiling scores but not considering their wrist to be normal [72].

From the cost-effectiveness perspective, casting is the most cost-effective treatment modality in the elderly with closed extra-articular DRFs. As to unstable ones, percutaneous pinning is more cost-effective than VLPF or EF [73].

### 2.4. Complications following Treatment for Elderly DRFs

In a retrospective analysis of patients above 65 years of age with 13,713 DRFs, the most common 1-year upper-extremity-specific complication is post-injury stiffness (11.5%), which is significantly more frequent following surgical managements than non-surgical treatments (16.0% versus 9.8%). There is no significant difference between surgical and non-surgical managements in terms of 90-day complication rates. However, surgical managements have a higher 1-year complication rate than non-surgical managements. Overall, the five most common upper-extremity-specific complications following surgical treatments of DRFs are stiffness (16.0%), CRPS (9.9%), median neuropathy (8.0%), implant-related complications (3.8%), and tendon-related complications (2.8%) [9]. Among implant-related complications, VLPF might lead to tenosynovitis, implant-associated pain, and screw protrusion, so regular monitoring and assessment of possible complications that warrant further implant removal are crucial within 3 years post-surgically [74].

In the WRIST trial, 18.5% of patients in the ORIF group report a median nerve compression, while 25.8% receiving EF and 23.2% receiving percutaneous pinning sustained pin site infections. Compared to ORIF, the rate of complications with any severity is higher in those receiving casting, whereas the rate for moderate complications is higher from EF [43,75].

### 2.5. Post-Treatment Rehabilitation

The efficacy of rehabilitation programs such as hand therapies after treatments for DRFs has been uncertain; instead, they might place a transportation burden on patients and are costly on both individual and systematic levels. Nguyen et al. found that a hand strength-focused exercise program for elderly DRFs non-surgically managed with CI while significantly immobilized improves grip strength [76]. VLPF involving dissecting the pronator quadratus significantly lowers range of motion and pronation strength [77], so post-surgical rehabilitation might be beneficial. Nonetheless, Gamo et al. found that hand therapies improve the outcomes after VLPF for DRFs in middle-aged to elderly women at 8 weeks but not at 6 months after surgery [78]. The WRIST trial also concluded that hand therapies after DRFs may not be necessary for older patients [43,79]. Moreover, early mobilization after VLPF for DRFs does not lead to improved PROs [80].

### 2.6. Osteoporotic DRFs and Bone Cement Augmentation

Osteoporosis is a predominant factor for low-energy DRFs in the elderly, and osteoporotic DRFs occur at a younger age than other fragility fractures. Hence, it might be the first opportunity to evaluate and treat osteoporosis and to prevent future secondary fragility fractures, such as vertebral, hip, and proximal humeral and femoral fractures [22,81]. Treatments of osteoporotic DRFs are two-fold, involving both the orthopedic intervention and more importantly the management of metabolic diseases. Non-pharmacologic treatments consist of physical therapy, fall prevention, smoking and alcohol cessation, dietary modification, and vitamin D supplementation [23,82]. The first-line pharmacologic therapy is bisphosphonates with an evident decrease in future osteoporotic fractures and safety for immediate use following fractures [23,82,83]. Nevertheless, a Swedish national register study found that osteoporosis medication for secondary fracture prevention remains low despite suggestion from current guidelines [84].

As an attempt to improve fracture stability and ultimate healing, the use of biomaterials for augmentation of osseous voids and fracture fixation is a promising treatment option. Augmentation techniques can be applied in various locations, and fractures of the metaphyseal regions, such as the proximal humerus, femur, tibia, and distal radius, remain the most common areas for their use. So far, one of the most widely utilized bone graft substitutes is calcium phosphate bone cement (CPBC). Calcium phosphate mimics the mineral phase of bone with osteo-conductivity and gradual remodeling (i.e., osteo-inductivity) over time, and achieves compressive strength greater than normal cancellous bone within a few minutes in the injectable form [21].

## 3. VLPF with BCA for DRFs

Given that VLPF alone is unable to guarantee superior outcomes to conservative treatments, orthopedic surgeons started wondering whether the addition of BCA could overcome the demerits of using a VLP alone. In this section, we reviewed the available literature regarding the use of VLPF with BCA for DRFs, especially in the elderly. According to our survey, there have been merely six related studies to date, including one RCT [24], three retrospective studies [25,26,85], and two biomechanical studies [27,28] (Table 2). We summarized their main findings with our appraisals in the following sections.

### 3.1. Randomized–Controlled Trial

Kim et al. conducted an RCT to evaluate whether VLPF with CPBC augmentation (CPBCA) had any benefit over VLPF alone in patients older than 65 years with unstable DRFs [24]. Overall, 48 patients involving 50 DRFs were recruited. There were 40 female patients and 8 male patients with a mean age of 73 years (range, 65 to 89 years). A total of 29 fractures were in the right wrist. After randomization, all of the surgical procedures were performed by one orthopedic surgeon. In the VLPF group, 25 DRFs were stabilized with VLPs alone; in the VLPF with CPBCA group, 25 DRFs were stabilized with VLPF and addition of injected CPBC through cortical defects on the radial side of DRFs while metaphyseal defects were directly visualized. A short arm splint was applied and worn for 2 weeks after surgery for both groups, and a removable short arm brace was used but removed for active wrist motion exercises during weeks 2 through 4. At 3 and 12 months post-surgically, no significant difference was found between the two groups for any clinical parameter (i.e., mean range of motion (flexion arc, extension arc, supination arc, and pronation arc), grip strength, VAS scores, modified MWS (MMWS), and DASH scores), and no significant difference was observed between groups with regard to any radiographic parameters in the initial post-surgical period and at 12 months post-surgically. Post-surgical complications (e.g., loss of reduction, surgical site infection, tendon-related complications, etc.) were rare in both groups. However, an obvious limitation of this study is that all of the surgical procedures were performed by only one orthopedic surgeon in a medical center, and he was informed of whether CPBCA was required at the start of the surgery instead of after VLPF. Moreover, the sample size was quite small and might limit further conduction of subgroup analyses.

### 3.2. Retrospective Study

Mori et al. conducted a multicenter retrospective comparative study to compare clinical outcomes and cost-effectiveness of BCA on VLPF for unstable DRFs in the elderly [25]. A total of 485 patients were divided into the VLPF group and the VLPF with BCA group. After propensity score matching, the backgrounds of both groups were similar. The MMWS between groups was not significantly different. Radiographic evaluation revealed no implant failure in either group. Bone union was confirmed in all patients from both groups. The volar tilt, radial inclination, ulnar variance, and distal dorsal cortical distance values between the two groups were not significantly different. The mean total cost from VLPF with BCA was significantly higher than VLPF alone.

Furthermore, a retrospective comparative study by Chang et al. evaluated the necessity of BCA for DRFs fixed with VLPF [26]. Overall, 105 fractures were included and divided into the non-BCA group (*n* = 88) and the BCA group (*n* = 17). Of the 105 fractures, 54 were identified as comminuted types according to the AO classification (A3, C2, and C3), and similar radiographic outcomes were noted. Within each group, both groups exhibited significant differences in dorsal collapse and radial height shortening, but volar tilting and radial inclination did not differ significantly. There was no difference in the degree of dorsal collapse and radial height shortening between the two groups. However, not only elderly patients but also younger patients with a lower prevalence of osteoporosis were included, which might limit its clinical applicability to elderly DRFs. Moreover, neither of the above two studies provided detailed information regarding the types of bone substitutes used.

Nonetheless, in a study by Goto et al. investigating the benefit of augmentation for maintaining a fracture reduction, elderly patients suffering from DRFs with metaphyseal comminution were treated by using a fixed-angle VLP with or without CPBCA [85]. There was no significant difference between the two groups in terms of volar tilt and radial inclination; however, ulnar variance increased significantly in the group treated with VLPF alone. Consequently, it might be useful to use a combination technique of VLPF with CPBCA to treat comminuted DRFs in elderly patients.

### 3.3. Biomechanical Study

In a biomechanical study through a cadaveric model by Högel et al., AO-type 23-A3.3 fractures were made in eight pairs of fresh-frozen osteoporotic cadaveric radial bones, and CPBC was used for augmentation. All specimens were treated with VLPF and averagely divided into the BCA or the non-BCA groups. The authors found that BCA resulted in a significant increase in cycles and load to failure. Compared to the non-BCA group, fracture gap movement decreased significantly at loads higher than 325 N, as did screw cutting distance at the holes of the ulnar column in the BCA group. Hence, CPBCA might improve biomechanical properties in VLPF of DRFs [27].

Similarly, Kainz et al. used human fresh-frozen cadaver pairs of the radius to simulate an AO/OTA 23-A3 fracture. In a total of four groups (*n* = 7 for each group), two kinds of volar fixed-angle plates with or without an additional injection of CPBC into the dorsal comminution zone were used to fix the distal metaphyseal fragment. Improved biomechanical properties (e.g., decreased displacement, increased stiffness, and decreased dissipated work) were found in plates if CPBC was additionally injected. Injection of CPBC into the dorsal comminution zone increased stability after VLPF of DRFs in vitro [28].

### 3.4. Our Experiences

**Figure 1:**
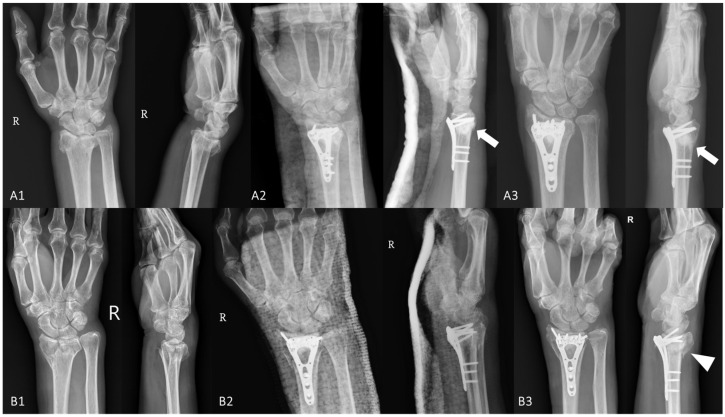
Comparison of volar locking plate fixation (VLPF) (**A1**–**A3**) with or (**B1**–**B3**) without calcium phosphate bone cement augmentation (CPBCA) in elderly distal radial fractures (DRFs). Patient A with (**A1**) a right DRF underwent (**A2**) VLPF with CPBCA (arrows) and received (**A3**) a radiologic follow up at 6 months post-surgically; similarly, patient B also with (**B1**) a right DRF underwent (**B2**) VLPF without CPBCA and received (**B3**) a radiologic follow up at 6 months post-surgically. Compared to patient A treated with VLPF with CPBCA (arrows), gradually decreased radial height, loss of volar tilting, and dorsal migration of DRF fragments (arrowhead) were noted in patient B treated with VLPF without CPBCA.

In our medical center, we retrospectively analyzed elderly patients aged above 65 years who presented with a DRF due to a low-energy injury and who were subsequently treated with VLPF (Variable Angle LCP^®^, DePuy Synthes^TM^, Raynham, MA, USA) with CPBCA (Pro-Dense^®^, Wright Medical Technology^TM^, Arlington, TN, USA) from October 2022 to June 2023. Those who had pre-existing severe comorbidities, an ipsilateral upper extremity injury, a previous wrist injury, surgical delay for more than 2 weeks, and an insufficient quantity of injected CPBC less than 1 milliliter were excluded. After excluding 4 patients aged younger than 65 years and 2 patients with radiologic dorsal cortex defects, a total of 18 patients, including 8 men (mean age: 78.5 years; range, 66–94) and 10 women (mean age: 88.6 years; range, 82–102), were included in the analysis. There was no intra-operative complication noted, and immediate post-surgical radiographs demonstrated anatomic reduction in 15 patients and near-anatomic reduction in 3 patients. At post-surgical 3-month and 6-month follow ups, there was no fixation failure (e.g., screw cut-outs or loss of fixation), and the mean VAS scores were low (1.27, 0 to 8; 0.44, 0 to 3). No patient complained of a decreased range of wrist motion and function in physical activities. However, two patients had a minor secondary plate/screw displacement due to compression to the fracture site, but surgical revision was not necessary in either of them. In addition, no post-surgical complication (e.g., nonunion, surgical site infection, tendon-related complication, etc.) was observed during the follow ups. Compared to the first post-surgical radiograph, follow-up radiologic assessment revealed good improvement in radial height, radial inclination, and volar tilt among all patients. As aforementioned, CPBC with osteo-conductivity and osteo-inductivity similar to mineral bones could augment the osseous voids in osteoporotic bones [21]. Among the elderly with a higher prevalence of osteoporosis and further risk of unstable or comminuted DRFs induced with osteoporosis, VLPF alone may not provide sufficient structural support to maintain post-surgical radial height and inclination, especially in patients with significant intra-articular or metaphyseal bone loss due to severe osteoporosis; however, in our experiences, CPBCA could provide additional biomechanical support to prevent subsequent collapse and promote bone healing. As shown in Figure 1, we indeed observed that the addition of CPBCA to VLPF maintains better anatomical configuration and decreases loss of fixation, delayed union, and nonunion.

## 4. Summary

To the best of our knowledge, this is the first article focusing on comprehensively reviewing the evidence of utilizing VLPF with BCA in the treatment of elderly DRFs, which are not covered in the two most recent review articles about geriatric DRFs in detail [5,86]. However, due to the limited number of studies with almost unanimous outcomes investigating this treatment modality in vivo [24,25,26,85], a meta-analysis with sufficient power is infeasible [87].

Based on our review, conservative non-surgical treatments evidently result in non-inferior clinical and functional outcomes, and complication rates compared to surgical treatments for elderly DRFs, but surgical treatments can yield a faster recovery to previous levels of activity in appropriately selected elderly and super-elderly patients. Applying shared decision making and the 2020 DRF AAOS/ASSH CPG may aid and assist orthopedic surgeons in choosing individually suitable treatments [15,88,89]. Furthermore, the use of minimally invasive procedures in treating elderly DRFs might become a trend in the future but requires further research. As to post-surgical outcome measurements, the following are reported outcome prediction factors in elderly DRFs: pain at enrollment, education, physiological age, number of comorbidities, ulnar positivity greater than 2 mm, persistent articular gaps/steps greater than 2 mm, and pre-injury activities. Moreover, rehabilitation with hand therapies can facilitate faster recovery of grip strength after treatment for elderly DRFs. Lastly, post-injury stiffness, CRPS, median neuropathy, implant-related complications, and tendon-related complications are among the five most common upper-extremity-specific complications following surgical treatments of DRFs. More importantly, regarding the focus of the present review, VLPF with BCA results in better biomechanical outcomes in vitro but non-superior PROs for elderly DRFs in vivo, probably with uncaptured associated factors between the gap to be identified. We suggest that a thorough evaluation should be performed according to each elderly patient’s individualized condition before using VLPF with BCA.

Due to the limited number of studies with inconsistent types of bone cement used, further RCTs with a larger sample size investigating the effectiveness of VLPF with BCA for elderly DRFs are still warranted to clearly verify whether this combination surgical procedure could contribute to better outcomes or instead lead to unnecessarily increasing medical costs. We also suggest that subgroup analyses according to factors associated with DRFs, such as age, gender, activity levels, comorbidities like osteoporosis, AO classification, etc., should be conducted in further studies to identify certain sub-populations that could benefit more from VLPF with BCA. Moreover, PRO assessment tools with less ceiling effect and predictive models to determine whether elderly patients with DRFs should undergo conservative or surgical treatments deserve future research by integrating the associated risk and predictive factors with elderly DRFs, thereby enhancing the efficiency of medical resource distribution simultaneously in accordance with patients’ preferences and expectations. Lastly, more studies regarding the use of minimally invasive procedures in treating elderly DRFs are anticipated.

Our review has several limitations. First, a systemic review and meta-analysis was not performed following the Preferred Reporting Items for Systematic Reviews and Meta-Analyses (PRISMA) guideline [90], so the credibility might be finite. However, this review focuses on VLPF with BCA for elderly DRFs, and there have been a limited number of associated studies as mentioned above. Hence, the present study still provides the most comprehensive review regarding VLPF with BCA for elderly DRFs to date. Second, although we seek to offer an updated review, some related studies might be published after the publication of this article. Third, due to a lack of associated studies with discordant research designs as aforementioned, we still could not offer an affirmative suggestion of using VLPF with BCA for elderly DRFs. Nevertheless, VLPF with BCA seems to be an effective and harmless treatment modality according to our clinical experiences.

In conclusion, surgical treatments yield superior short-term but comparable long-term clinical and functional outcomes compared to non-surgical treatments for elderly DRFs. Further studies are required to determine the effectiveness of VLPF with BCA, develop better assessment and predictive tools, and validate the utilization of minimally invasive procedures for elderly DRFs.

## Figures and Tables

**Table 1:** Patient-reported outcome measure (PROM) tools commonly used to evaluate treatment effectiveness of distal radius fractures (DRFs).

Modified Mayo Wrist Score (MMWS) [56,57]	Require both patient and physician participation to assess pain, active flexion/extension arc (in comparison with the contralateral side), grip strength (in comparison with the contralateral side), and the ability to return to regular employment or activities. Scores range from 0 to 100 with a score of 0 indicating the worst wrist condition and 100 indicating the best wrist condition.
Patient-Rated Wrist Evaluation (PRWE)/Patient-Rated Wrist/Hand Evaluation (PRWHE) [58,59,60]	A self-administered, patient-specific questionnaire consisting of 2 subscales with 15 items designed to measure wrist pain and disability in activities of daily living. The pain subscale contains 5 items with each item rated from 1 to 10. The function subscale contains 10 items divided into 2 sections (i.e., specific activities (6 items) and usual activities (4 items)). The maximum score in both sections is 50 and minimum is 0. A score of 100 represents the worst functional score, whereas 0 represents no disability.
Disabilities of the Arm, Shoulder, and Hand (DASH) score [60,61]	A 30-item self-reported questionnaire in which the response options are presented as 5-point Likert scales. Scores range from 0 (no disability) to 100 (the most severe disability). This score was designed to be useful in patients with any musculoskeletal disorder of the upper limb.
QuickDASH [62,63]	A subset of 11 items from the 30-item DASH and is a self-reported questionnaire in which the response options are presented as 5-point Likert scales. At least 10 of the 11 items must be completed for a score to be calculated and the scores range from 0 (no disability) to 100 (the most severe disability). This score was designed to be useful in patients with any musculoskeletal disorder of the upper limb.

**Table 2:** Studies investigating the effectiveness of volar locking plate fixation (VLPF) with or without bone cement augmentation (BCA) for distal radius fractures (DRFs).

Study	Study Type	Subjects (*n*)	Intervention (*n*)	Comparator (*n*)	Outcome Measurements	Main Results	Conclusion
Kim et al. [24]	Randomized–controlled trial	48 patients (40 females and 8 males) involving 50 DRFs with a mean age of 73 years (range, 65 to 89 years)	VLPF with CPBCA (*n* = 25)	VLPF alone (*n* = 25)	Clinical outcome: Grip strength, wrist range of motion, subjective wrist pain (VAS scores), MMWS, and DASH scores	No significant difference was observed between groups regarding clinical and radiologic outcomes. No complication-related difference was observed, and there was no nonunion.	VLPF with CPBCA of metaphyseal defects offered no benefit over VLPF alone in elderly patients with unstable DRFs.
					Radiologic outcome: RI, VT, and UV		
Mori et al. [25]	Retrospective comparative study	485 patients aged 65 years or older	VLPF with BCA (*n* = 97)	VLPF alone (*n* = 388)	Clinical outcome: MMWS	The MMWS, VT, RI, UV, and DDD values between groups were not significantly different. Radiologic evaluation revealed no implant failure in either group. Bone union was confirmed in all patients in both groups. The initial surgery cost and total cost from VLPF with BCA were significantly higher than those from VLPF alone (USD 3515 vs. USD 3068, *p* < 0.001)	The clinical and radiologic outcomes of VLPF with BCA did not differ from those of VLPF alone, yet the additional use of BCA was associated with higher medical costs.
					Radiologic outcome: Implant failure rate, bone union rate, VT, RI, UV, and distal DDD		
					Cost-effectiveness evaluation: Initial surgery cost and total cost		
Chang et al. [26]	Retrospective comparative study	105 patients (60 females and 45 males) with an average age of 50.8 years (range, 19 to 83)	VLPF with BCA (*n* = 17)	VLPF alone (*n* = 88)	Radiologic outcome: RH, RI, VT, and dorsal collapse	Both groups exhibited significant differences in dorsal collapse (*p* < 0.001 and *p* = 0.001, respectively) and radial height shortening (*p* < 0.001 and *p* = 0.039, respectively); VT and RI did not differ significantly. There was no difference in the degree of dorsal collapse (*p* = 0.715) and radial height shortening (*p* = 0.651) between the 2 groups.	VLPF is an effective and reliable treatment method without additional BCA needed even for comminuted DRFs if sufficient anatomical reduction fixation is achieved
Goto et al. [85]	Retrospective study	24 patients (18 females and 6 males) involving 25 DRFs with an average age of 64.9 years (range, 50 to 86 years)	VLPF with CPBCA (*n* = 7)	VLPF alone (*n* = 18)	Radiologic outcome: VT, RI, and UV	No significant difference between the 2 groups in terms of VT (*p* = 0.80), RI (*p* = 0.17), grip strength (*p* = 0.60), and Saito scores (*p* = 093), but UV increased significantly in the group treated with VLPF alone (*p* < 0.05).	It might be useful to use a combination technique of VLPF and CPBCA to treat comminuted DRFs in elderly patients.
					Clinical assessment: Grip strength and range of wrist flexion/extension, radial/ulnar deviation, and supination/pronation		
					Clinical outcome: Saito scoring system		
Högel et al. [27]	Biomechanical study	8 pairs of fresh-frozen osteoporotic cadaveric radius with AO-type 23-A3.3 fractures	VLPF with CPBCA (*n* = 8)	VLPF alone (*n* = 8)	Load to failure, construct stiffness, fracture gap movement, and screw cutting distance	CPBCA resulted in significant increases in cycles, load to failure, and construct stiffness at loads higher than 325 N, and fracture gap movement and screw cutting distance decreased significantly at this load and higher compared to VLPF alone.	CPBCA improved biomechanical properties in VLPF for DRFs.
Kainz et al. [28]	Biomechanical study	14 pairs of human fresh-frozen cadaveric radius with AO/OTA-type 23-A3 fractures	VLPF with CPBCA (*n* = 14)	VLPF alone (*n* = 14)	Displacement, stiffness, dissipated work, and failure mode	Decreased displacement, increased stiffness, and decreased dissipated work were found in VLPF with CPBCA. Pushing out of the screws was noticed as a failure mode only in samples lacking supplementary biomaterial.	CPBCA of dorsal comminution zone increased stability after VLPF for DRFs in vitro.

DRF: Distal radius fracture; VLPF: Volar locking plate fixation; CPBCA: Calcium phosphate bone cement augmentation; VAS: Visual Analogue Scale; MMWS: Modified Mayo Wrist Score; DASH: Disabilities of the Arm, Shoulder, and Hand; RI: Radial inclination; VT: Volar tilt; UV: Ulnar variance; DDD: Dorsal cortical distance; BCA: Bone cement augmentation; RH: Radial height.

## Data Availability

Not applicable.
